# The discovery of how gender influences age immunological mechanisms in health and disease, and the identification of ageing gender-specific biomarkers, could lead to specifically tailored treatment and ultimately improve therapeutic success rates

**DOI:** 10.1186/1742-4933-9-24

**Published:** 2012-11-13

**Authors:** Anna Maria Berghella, Ida Contasta, Tiziana Del Beato, Patrizia Pellegrini

**Affiliations:** 1 Consiglio Nazionale delle Ricerche (CNR), Istituto di Farmacologia Traslazionale (IFT) via G Carducci, 32 - Rotilio Center, 67100, L’Aquila, Italy; 2 Istituto di Farmacologia Traslazionale Consiglio Nazionale delle Ricerche via G Carducci, 32 - Rotilio Center, 67100, L'Aquila, Italy

## Abstract

The control of human health and diseases in the elderly population is becoming a challenge, since mean age and life expectation are progressively increasing as well as chronic degenerative diseases. These disorders are of complex diagnosis and they are difficult to be treated, but it is hoped that the predictive medicine will lead to more specific and effective treatment by using specific markers to identify persons with high risk of developing disease, before the clinical manifestation. Peripheral blood targets and biomarkers are currently the most practical, non-invasive means of disease diagnosing, predicting prognosis and therapeutic response. Human longevity is directly correlated with the optimal functioning of the immune system. Recent findings indicate that the sexual dimorphism of T helper (Th) cytokine pathways and the regulation of Th cell network homeostasis are normally present in the immune response and undergoes to adverse changes with ageing. Furthermore, immune senescence affects both men and women, but it does not affect them equally. Therefore, we hypothesize that the comprehension of the interferences between these gender specific pathways, the ageing immunological mechanism in pathological or healthy state and the current therapies, could lead to specifically tailored treatment and eventually improve the therapeutic success rates. Reaching this aim requires the identification of ageing gender-specific biomarkers that could easily reveal the above mentioned correlations.

## Background

The progressive increase of mean age and life expectation are correlated to a rise of chronic degenerative diseases such as cancer, cardiovascular, autoimmune or neurodegenerative diseases among the elderly population and these changes will challenge our ability to manage human health and diseases of this category. To this aim, researchers are conducting programs to better understand human ageing and ageing-related diseases and dysfunction. Chronic degenerative diseases are of complex diagnosis, they are difficult to be treated and they absorb an increasing proportion in the health care budgets worldwide, a phenomenon that will bring social, political, economic and biomedical challenges to future generations [1]. However, recent developments in modern medicine, especially in genetics, proteomics, and informatics, are leading to the discovery of biomarkers that can be used as indicator of disease’s risk in healthy subjects.

Predictive medicine uses markers to identify persons with high risk of developing disease before the clinical manifestation. It is hoped that this approach will lead to more specific and effective treatment in the not too distant future but this success depends upon the identification of specific biomarkers that can be measured easily and early, from disease onset. Peripheral blood targets and biomarkers are currently the most practical, non-invasive means of disease diagnosing, predicting prognosis and therapeutic response [2].

The identification of ageing gender-specific pathways and biomarkers in peripheral blood would therefore open up an interesting field for research in human health and disease, since gender is related to disease susceptibility [3] and these pathways suffer adverse changes with ageing. Researchers have been shown that sex steroids, for example, influence the regulation of Th cell network balance, shifting the balance toward a Th1 and/or Th2 type response and both clinical and experimental data have demonstrated the presence of a natural sexual dimorphism in the immune response [3-6]. During their reproductive years, females have a more vigorous cellular and humoral immune response than males and they also have a greater ability to reject tumors and homografts [7-12]. Furthermore, immunosenescence affects both men and women, but it does not affect them equally. Men (all ages) and postmenopausal women exhibit diminished T cell immunity compared to premenopausal women [13]. The decrease in androgens in men with ageing may contribute to their immunosenescence; however, the loss of T cell function in men with ageing is significantly less dramatic than what has been observed in women [14-16]. There are multiple forms of estrogen: estrone (E1), estradiol (E2) and estriol (E3) are the primary circulating forms. Estradiol binds both estrogen receptor-(ER)α and ERβ with high and equal affinities, while estrone preferentially binds ERα at a 5-fold higher affinity than ERβ [17]. However, both pathways are involved in mediating estrogen effects, but ERα and ERβ exhibit distinct functions within immune cells [18]. In premenopausal women ovary-derived estradiol is the principal circulating estrogen, while estrone is the most abundant circulating estrogen in postmenopausal women and men. In men, testosterone is the primary substrate for estrogen production by peripheral aromatization of androgens precursors, but exhibits a small age-related decrease. Furthermore, most studies failed to observe any significant influence of age on total E2 levels in men [16].

### Presentation of the hypothesis

Therefore, the comprehension of how and why immune responsiveness changes in humans with ageing is essential for developing strategies to prevent or restore deregulated immunity and assure healthy longevity. Here, we advance the hypothesis that the discover of how gender influences age immunological mechanisms in health and disease, and the identification of ageing gender-specific biomarkers, could lead to specifically tailored treatment and ultimately improve therapeutic success rates. We discuss published data on gender-dependent immune responses in health and disease states.

It is well established that the gender of a host can significantly affect susceptibility to infection [19]. Epidemiological clinical data and animal models of various human illnesses including sepsis [20] and listeriosis [21,22] reveal that males and females handle infections differently and gender-dependent immune pathway or molecules causes different disease susceptibility in men and women (Table 1).

**Table 1:** Gender-dependent immune pathways or molecules that causes different disease susceptibility in men and women

**Gender dependent immune pathways or molecules**	**Disease susceptibility**	**References**
**Caspase-12 long variant**	infectious diseases	Yeretssian G, Proc Natl Acad Sci, 2009, **106:**9016
**Glucocorticoid receptor pathways**	infectious diseases	Wynne O, Stress, 2011, **14:**247
**Sulfur-dependent detoxification pathways**	autism	Al-Yafee YA, BMC Neurol, 2011, 11:139
**MyD88-dependent pathway**	type 1 diabetes	Sheng H J, Immunol, 2011, 187:1591
**STAT5-dependent pathway**	human growth disorders	Davey HW, Am J Hum Genet, 1999, 65:959
**Toll-like receptor 4**	chronic pain	Sorge RE, J Neurosci, 2011, 31:15450
**B7-H1-dependent pathway**	melanoma	Lin PY, J Immunol, 2010, 185:2747
**PARP-1-dependent pathway**	lupus nephritis	Caricchio R, NIH RePORTER, 2012, 08 01
HLA-DRB1*0401 and HLA-DQ8	arthritis	Behrens M, J Autoimmun, 2011, 37:95
**Rac1, NOX, ROS**	kaposi sarcoma	Goldschmidt CPJ, NIH RePORTER, 2010, 05 01
**CTLA4**	hepatitis C	Schott E, J Hepatol. 2007, 46:372
**Toll-like receptor 7**	chronic HCV-infection	Schott E, J Hepatol, 2007, 47:203
**Aim2 and p202 proteins**	systemic lupus erythematosus	Panchanathan R, Mol Immunol, 2011, 49:273
**Retinoid x receptor alpha**	hepatocellular carcinogenesis	Guo M, BMC Genomics, 2008, 9:403
**MAPK signaling**	systemic lupus erythematosus	Xie H, Arthritis Rheum; 2011, 63:2425

Women have a more powerful immune system than men: the production of estrogen by females could have a beneficial effect on the innate inflammatory response against bacterial pathogens. Inflammatory caspases, for example, are important effectors of innate immunity [23] and the activity of caspase-1 is regulated by related inflammatory caspases, namely caspases-5 and -11, which activate select inflammasomes [24,25], and caspase-12, which represses caspase-1 catalysis [26]. Caspase-12 is expressed in all mammals tested to date, but has acquired deleterious mutation in humans [26]. A single-nucleotide polymorphism introduces a premature stop codon in caspase-12 in the majority of the population. In a study, a fully humanized mouse that expresses the human caspase-12 rare variant (Csp-12 L) in a mouse casp-12-/- background, was generated and the modalities by which human caspase-12 confers susceptibility to infection [27] was examined. In this study, an unsuspected hormonal regulatory mechanism that governs human Csp-12 L expression during infection, has been identified. These results indicate that through estrogen production, females have a built-in mechanism that prevents Csp-12 L from being expressed, favoring more robust inflammatory and immune responses to pathogens [27].

Sex steroids influence the regulation of Th cell network balance, shifting the balance toward a Th1 and/or Th2 type response. In healthy men and women, the polarization of immune response into TH1 or TH2 cytokines or cellular types is not absolute and the ratio of these cells varies according to physiological demand and clinical conditions [28]. In vivo and in vitro studies in mice have demonstrated that cytokines play a crucial role in maintaining balance in the differentiation of T cells into TH1 or TH2 types [29]. It was found that pathological conditions arise from abnormalities in the balance between the production of TH1 and TH2 cytokines [30]. It seems that the relative proportion of each cell type depends on the environmental cytokines present during activation [31].

The sex hormones may affect the Th1/Th2 balance, in man and women. However, if we consider the susceptibility difference to autoimmune diseases and infectious diseases between man and women, the change in the profile of cytokines could be the result of the adaptive response, rather than the cause of the different susceptibility. This implies that hormone-dependent mechanisms operate in the selection of cell phenotypes that regulate the adaptive response and which, if altered, may affect the balance of the Th cells. Induction of Treg cells by estrogen physiological level was, for example found [32]. These author conclude that estrogen is a potential physiological regulatory factor for the peripheral development of CD4+CD25+ Treg cells [32]: estrogen receptor exist on the CD4+CD25- T cells and the conversion of CD4+CD25- T cells into CD4+CD25+ Treg cells is stimulated by estrogen. Additionally, it was discovered that hormones peripherally activated pro-hormones and regulated the Th1/Th2 balance [33].

There is evidence that sex hormones can affect the immune system and that female and male hormones act in opposing ways [34,35]. For example, Th1 and Th2 responses appear affected by androgenic and estrogenic preponderance, respectively: androgens favor the development of a Th1 response and activation of CD8 cells [36], while estrogens seem to direct the immune system towards Th2 dominance, where B lymphocytes are activated and antibody production flourishes [35]. Pregnancy, a high estrogen state, is of course characterized by Th2 preponderance, and a failure in the establishment of the Th2 dominance has been associated with increased risk for pregnancy loss [37,38].

Gender accounts for important differences in the incidence and prevalence of a variety of age-related diseases. Research finding [39-41] points out that gender is a major variable in the genetics of longevity, suggesting that men and women follow different strategies to reach longevity. These results [41] confirm the age-related remodelling of cytokine network and that variations in pro- or anti-inflammatory cytokines might influence successful ageing and longevity, suggesting that the multiplex analysis of cytokine levels might be useful in defining a successful ageing profile [41,42].

**Figure 1:**
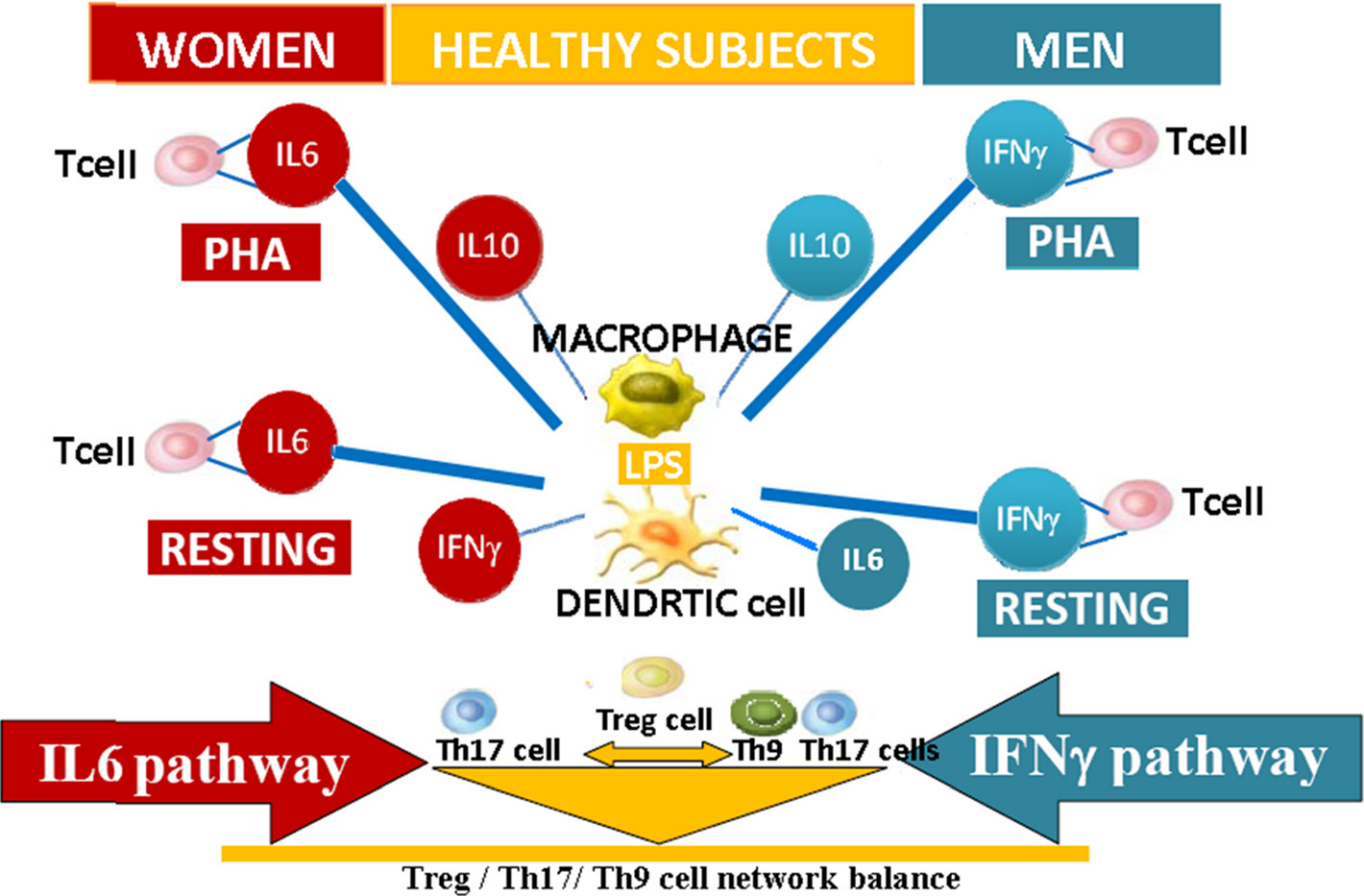
**Cytokine regulation of immune response cell phases through gender specific pathways.** Antigen presenting cells (APCs) regulate Th cell differentiation and Th cell network homeostasis under resting and activated conditions of the immune system in both men and women, however this effect appears to be exerted through male and female gender-specific health pathways: IL6 pathways regulate the homeostasis of the Th cell network in women, whilst this homeostasis is regulated by IFNγ pathways in men. The study [28] indicates that these regulatory differences do not usually have consequences until IFNγ and/or IL6 cytokine pathway alterations occur, ensuring the same result: a physiological homeostasis between Treg, Th17 and Th9 cells in the resting state, in the transition to the activation phase and in the return to the resting state.

A recent study [43] focusing on gender-dependent immune responses indicates, for the first time, that gender-specific Th cytokine pathways may well be responsible for the different gender-dependent responses to disease and therapy and open up an exciting new field for research. This study supports the concept that a sexual dimorphism of Th cytokine pathways and regulation of Th cell network homeostasis are normally present in the immune response. Antigen presenting cells (APCs) regulate Th cell differentiation and Th cell network homeostasis under resting and activated conditions of the immune system in both men and women, however this effect appears to be exerted through male and female gender-specific and gender-common health pathways (Figure 1).

IFNγ and IL-6 production pathways are the respectively male and female gender-specific health pathways for the immune response homeostasis and they are targets and/or biomarkers for the passage from health to adenoma and, eventually, to colorectal cancer. IL-10 pathway is the common-gender pathway which restores immune system resting homeostasis in both men and women, but only if it is controlled by the above mentioned gender specific pathways; otherwise IL10 pathway is a target/biomarker for cancer progression.

**Figure 2:**
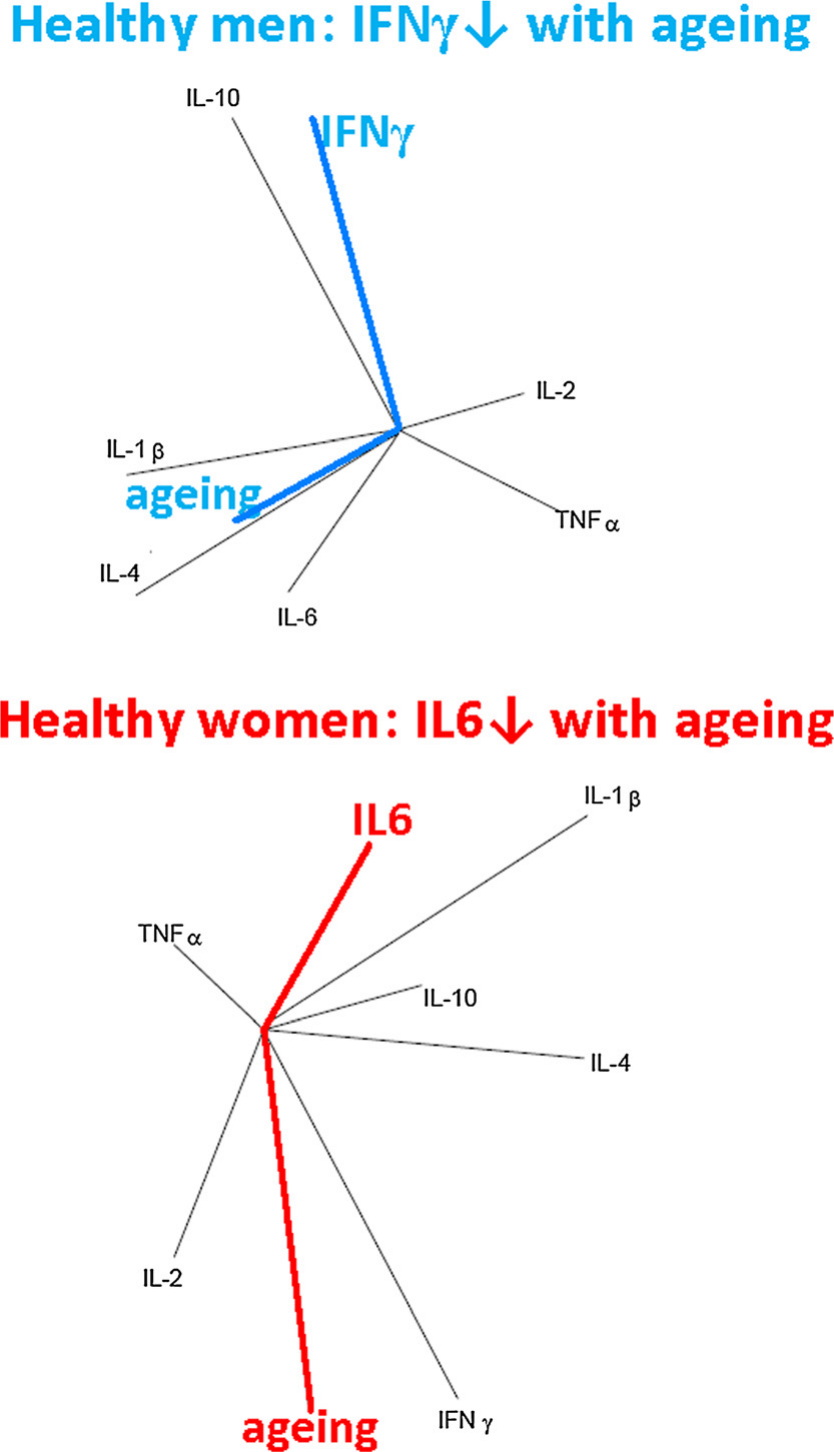
**The serum levels of IL6 in women and IFNγ in men decrease with increasing age.** A group of 66 healthy subjects of Italian people, 33 men and 33 women, (blood donors, laboratory staff and relative) were studied [28]. None of the subjects was receiving concurrent drug treatment including widely-used pharmaceuticals, such as salicylates and sex hormones (contraceptive pill, hormone replacement therapy). Distribution of age in male and female groups was the same (mean ± SD =41 ± 12 years, compared to mean ± SD = 41 ± 15 years, p = 0.14). In physiological systems components operate as a network and each component varies and co-varies dynamically with respect to one another. Therefore, the identification of physiological pathways, and correlated biomarkers can only be achieved through evaluations that take these fluctuations into account. Using the principal component analysis these authors plotted the network of vectors obtained by analyzing the data matrix of correlation/covariance coefficients of serum cytokines. In these plots, the angle between vectors is inversely proportional to the degree of correlation between vectors; the same vector direction indicates a positive correlation/covariance, the opposite vector direction indicates a negative correlation/covariance. This allows a visualization of the situation under study and is an excellent method for capturing significance from systems biology evaluations. The plots show that the serum levels of IL6 in women and IFNγ in men decrease with increasing age. The results of multiple regression analysis confirm that age could also be a significant independent factor for IFNγ (p = 0.01) and IL-10 (p = 0.03) in men; whilst in women age appears to be significant for sIL-6R (p = 0.002) and IFNγ (p = 0.04).

These findings showed that: i) the immune cell production of IL6 in women and IFNγ in men decreases with the increasing of the age (Figure 2); ii) age could also be a significant independent factor for IFNγ and IL-10 in men; iii) whilst in women age appears to be significant for IFNγ and the soluble interleukin 6 receptor (sIL-6R) molecule, which forms a ligand–receptor complex with IL6, that is capable of stimulating a variety of cellular responses including proliferation, differentiation and activation of inflammatory processes [44].

**Figure 3:**
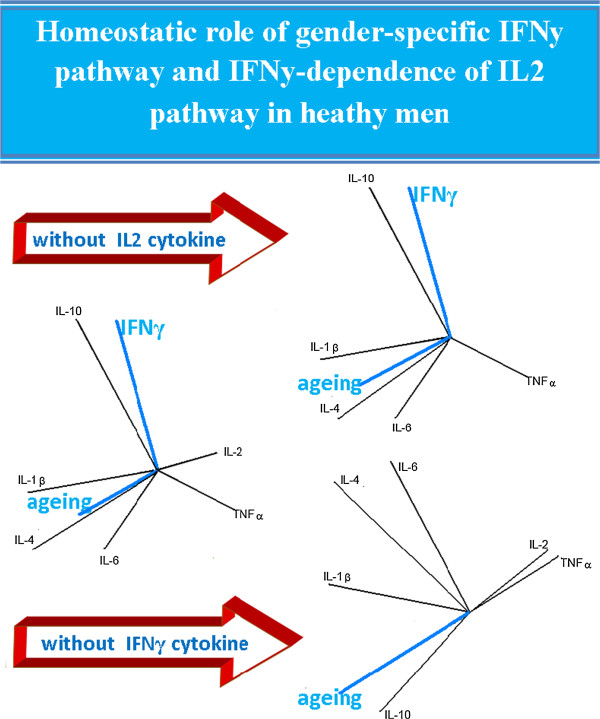
**Homeostatic role of IFNy pathway and IFNy-dependence of IL2 pathway in heathy men.** In the APC analysis, in heathy men (Figure 2) IFNγ level decreases with aging, however the IL-2 level seems to be a better candidate than IFNγ. The authors use their mathematical modeling to verify the IFNγ role as a gender-specific pathway, by excluding from the model IL2 and IFNy alternately. The graphs obtained from this APC analysis, clearly define the homeostatic role of IFNy and IFNy-dependence of IL2. Indeed, excluding IL2, reports from the network of cytokines are not affected, however the exclusion of IFNy completely reshapes these relationships.

Figure 2 shows that IFNγ level decreases with aging in healthy men, however the IL-2 level seems to be a better candidate than IFNγ level. To verify the IFNγ role as a gender-specific pathway, the authors use their mathematical modeling by excluding IL2 and IFNy cytokines alternately. The graphs obtained from the PCA analysis (Figure 3), clearly define the homeostatic role of IFNy and the IFNy-dependence of IL2. Indeed, excluding IL2 the relationships of the cytokine network are not affected, however the exclusion of IFNy completely reshapes these relationships.

**Figure 4:**
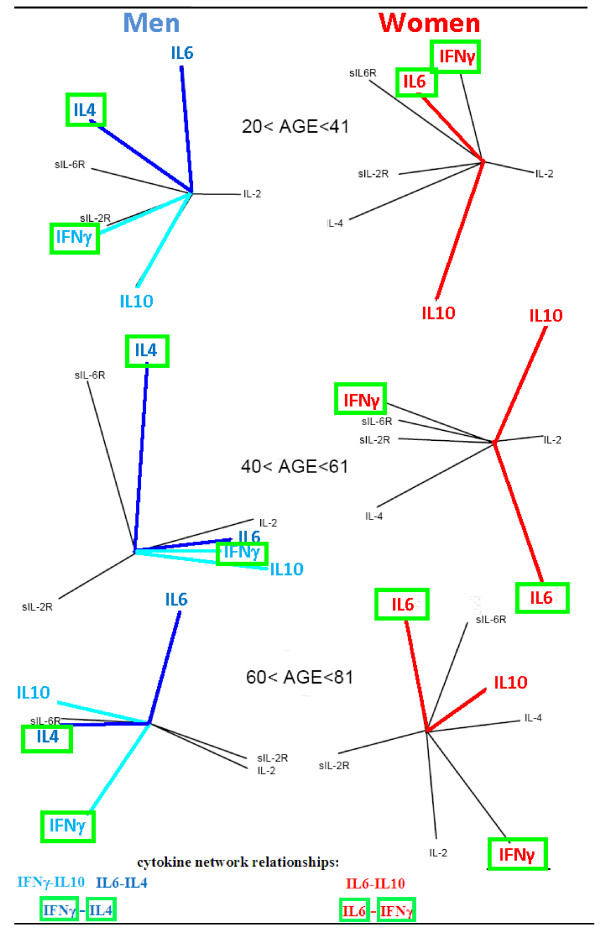
**Gender specific ageing variations in cytokine network relationships between pro- or anti-inflammatory cytokines could influence the success of the immune response.** The study results indicate that variations in specific cytokine network relationships, between pro- or anti-inflammatory cytokines, regulate immune response homeostasis in healthy state. The early evolution of immune response is controlled by the positive inter-regulation between production of **IFNγ-IL10** and **IL6-IL4** cytokines in men, and the negative inter-regulation of **IL6-IL10** cytokines in women. Similarly, the late evolution of immune response seems to be regulated by the positive inter-regulation between the production of **IFN**γ**-IL4** in men and by **IL6-IFN**γ in women. The principal component analysis has shown that these gender specific cytokine network relationships suffering changes during ageing, which could adversely affect the success of the immune response. Consequently, these cytokine relationships are dual gender specific biomarkers that could well be used to develop more specific approaches in the elderly population.

Gender specific variations in cytokine network relationships between pro- or anti-inflammatory cytokines influence the success of the immune response [41]. The results of the above mentioned study [43] showed that the early evolution of immune response is influenced by the positive inter-regulation between production of IFNγ-IL10 and IL6-IL4 cytokines in men, and the negative inter-regulation of IL6-IL10 cytokines in women. Similarly, the late evolution of immune response seems to be influenced by the positive inter-regulation between the production of IFNγ-IL4 in men and by IL6-IFNγ in women. These gender specific cytokine network relationships, between pro- or anti-inflammatory cytokines, vary with the increasing of the age (Figure 4) and they are dual gender specific biomarkers that could well be used to develop more specific approaches.

Overall, these findings underline the need for gender specific drugs that could take into account the different regulation system of the immune response, ensuring the same therapeutic result: the return to a physiological homeostasis thanks to the transition from a pathological activation phase to a physiological resting state. Obviously the regulatory differences do not usually have consequences until IFNγ and/or IL6 cytokine pathway alterations occur, in which case the consequences for men and women, in terms of pathological mechanisms and disease development, are different. The malfunctioning of gender specific pathways not only compromises the homeostasis of the immune response, but may also cause a pathological polarization of T cell subsets specific to each sex. In fact, IFNγ supports the development of Th1 functions [45] promoting cell mediated immunity, while IL6 cytokine supports Th2 responses where B lymphocytes are activated and antibody production flourishes. Additionally, research in this field has shown that it is not a single cytokine that determines a particular response but rather the interaction of individual cytokines within a network.

**Figure 5:**
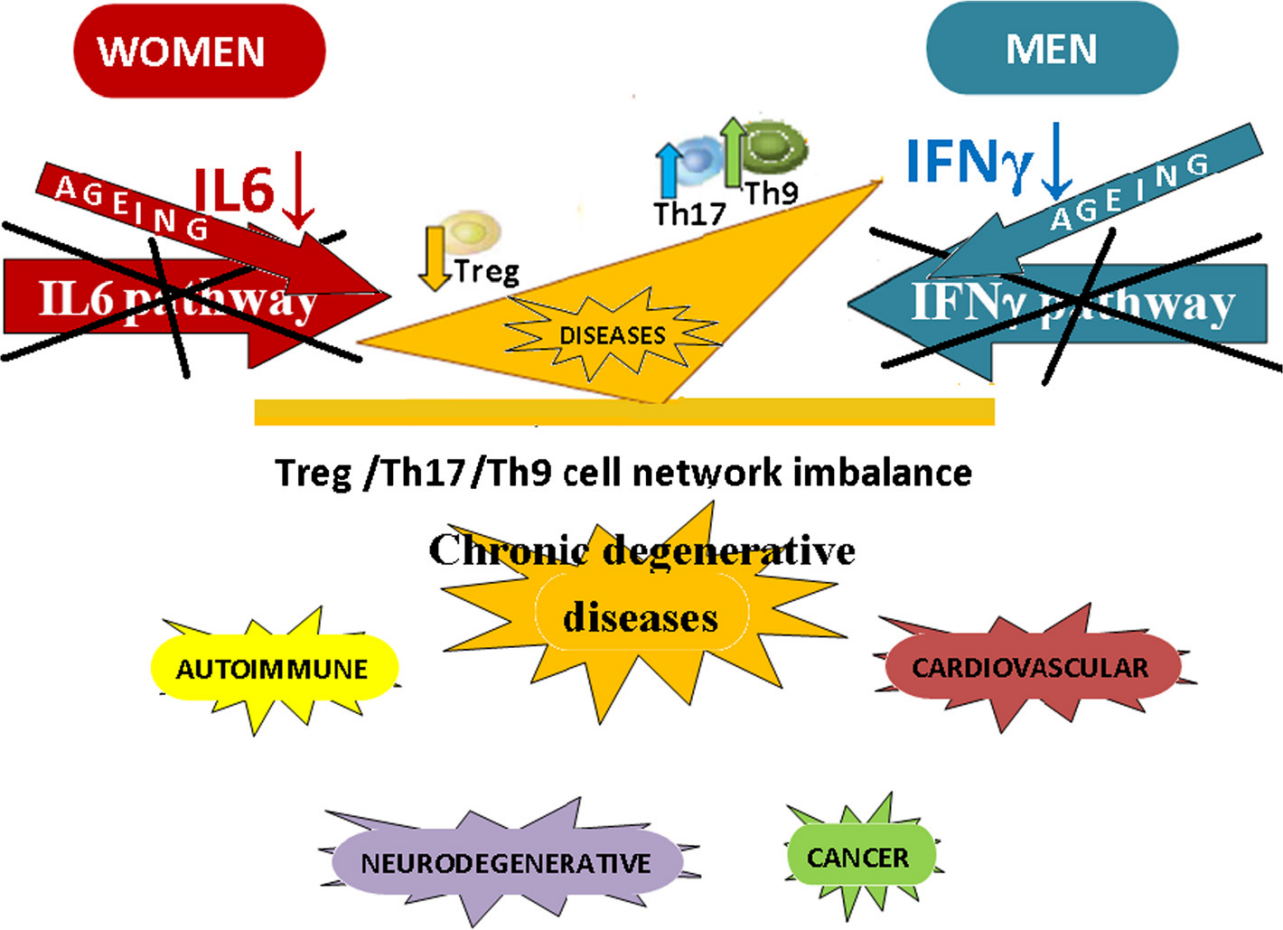
**A different gender susceptibility and clinical course in diseases during ageing could be caused by different Treg, Th17 and Th9 cell polarization.** IFNγ and IL6 cytokine pathway alterations occur with ageing, and the consequences for men and women, in terms of pathological mechanisms and disease development, are different because the malfunctioning of gender specific pathways not only compromises the homeostasis of the immune response, but may also cause a pathological polarization of T cell subsets specific to each sex. The results of the above mentioned study [28] indicate that a different gender susceptibility and clinical course in diseases during ageing is caused by different Treg, Th17 and Th9 cell polarization determined by the IFNγ and/or IL6 gender cytokine pathway interactions, which vary between men and women and with ageing [28]. Hence, autoimmune disease susceptibility in women could be attributed to the influence of ΙL6 which plays a key role in autoimmune diseases, such as multiple sclerosis, since it is a T cell differentiation switch factor for Tregs and Th17 cells. The greater likelihood of men developing the primary progressive multiple sclerosis form, on the other hand, could be the results of the influence of IFNγ on Th9 cell inhibition. Level variation of "dual gender specific cytokines" (for example IFNγ-IL10, IL6-IL4 and IFNγ-IL4 in men) in a direction that agrees with the positive or negative sign of their healthy relationship (in this case positive), it is an index for a transient inflammation, because it is still an indicator of immune system homeostasis; the level variation of "dual gender specific cytokines" in a direction that does not agree (in this case negative) with the positive or negative sign of their healthy relationship, it is index for a chronic inflammation, because it is an indicator of loss of homeostatic ability.

The results of the above mentioned study [43] indicate that a different gender susceptibility and clinical course in diseases is caused by different Treg, Th17 and Th9 cell polarization (Figure 5) determined by the TGFβ, IL6, IFNγ, ΖIL10 and IL4 cytokine pathway interactions which vary between men and women [43]. These findings are, indeed, backed up by the results of other researches indicating that there is a reciprocal development relationship between Treg, Th17 and Th9 cells because: i) TGFβ triggers the expression of Foxp3 transcription factor in naïve T cells, generating Treg cells, but ii) IL6 inhibits the TGFβ driven expression of Foxp3, and TGFβ together with IL6 induce ROR-gt transcription factor, triggering the developmental program of Th17 cells [46], while ii) IL4 also inhibits TGFβ induction of Foxp3 expression, but TGFβ together with IL4 induce the differentiation of Th9 cells, which produce IL9 cytokine. On the other hand, the co-expression of IL9 and IL17 was identified as a novel Th17 function in mediating immune and autoimmune tissue destruction [47,48], also in the central nervous system. Hence, autoimmune disease susceptibility in women, such as multiple sclerosis (MS), could be attributed to the influence of ΙL6, which plays a key role in autoimmune diseases, since it is a T cell differentiation switch factor for Tregs and Th17 cells [46,49-51]. The greater likelihood of men developing the primary progressive MS form [52], on the other hand, could be the results of the influence of IFNγ on Th9 cell inhibition. In fact, the IL9 receptor complex is constitutively expressed on astrocytes. IL9 induces astrocytes to produce CCL-20 but not other chemokines, including CCL-2, CCL-3, and CXCL-2 [53], suggesting that IL9 induces CCL-20 production by astrocytes to induce the migration of Th17 cells into the CNS. Treg, Th9 and Th17 cells have been shown to be important CD4 T cell subsets in human autoimmune diseases, including rheumatoid arthritis [54] and MS [55] diseases.

Our recent research on MS disease (article in press) confirms these data, by showing that a sexual dimorphism in autoimmune diseases is the result of different pathways that regulate the Th cell network homeostasis: IL6 pathways in women and IFNγ pathways in men. Since women are more susceptible to MS disease and the IL6 has a more significant role in the autoimmune process compared to IFNγ, it is logical to assume that IL6 pathways are in some way implicated in the prevalence of autoimmune diseases in women. Indeed, our data indicate that IL6 pathways are also involved in Treg cell imbalance and an increase in neurological deficit in both men and women groups of MS patients, underlining the autoimmune etiology of MS disease. In further support of differing cytokine pathways in men and women, we noted that the efficacy of IFNβ-treatment in the re-establishment of Th-network balance and delaying the progression of neurological disability is linked to the IL6 pathway in women, but to the IFNγ pathway in men.

The specific mechanisms responsible for gender specific disease susceptibility have yet to be clarified. However these data suggest that the answer may lie in the different capacity of cells to defend themselves against oxidative stress [56]. The cells of men and women differ greatly in terms of reactive oxygen species (ROS) production and oxidative stress susceptibility [57] and this appears to be a promising new field of investigation. Oxygen metabolism can lead to the production of ROS in all cell types. All cell types, including lymphocytes and other immune system cells, present antioxidant compounds and enzymes (such as glutathione and thioredoxin reductasi) [58,59] to neutralize ROS and to preserve the cell oxidative balance. However, the activities of ROS appear to be regulated differently in males and females and can be directly influenced by sex hormones [57].

In vivo studies have further demonstrated the incapacity in males, but not in females, of maintaining intracellular reduced redox conditions, essential for normal cellular functions [55], and this explains, at least in part, the differences between the two sexes in the maintenance of the immune system homeostasis. In fact, IFNγ is a direct stimulator of the gene expression of thioredoxin and thioredoxin reductase (RTrx) system in human T cells [59,60] and there is a positive feed-back circuit involving IFN-γ and Trx/RTrx gene expression in the regulation of intracellular reduced oxidative condition, which is essential for the immune response. Subsequently, we can assume that the immunological response through the IFNγ pathway in men reduces the intracellular oxidative levels to preserve the cell oxidative balance control. In fact, male cells, as we mentioned, are incapable of maintaining an intracellular reduced oxidative condition.

### Implications of the hypothesis

The comprehension of how and why immune responsiveness changes in humans as they age is essential for developing strategies to prevent or restore deregulated immunity and assure healthy longevity. Research findings showed that:

1. gender is a major variable in the genetics of longevity and men and women follow different strategies to reach longevity [39-41];

2. gender-specific Th cytokine pathways and the regulation of Th cell network homeostasis are normally present in the immune response [43]; IFΝγ and IL6 production pathways are the respectively male and female gender-specific health pathways for immune response homeostasis; IL10 pathway is the common-gender pathway which restores immune system resting homeostasis in both men and women, but only if it is controlled by the above mentioned gender specific pathways; these regulatory differences do not usually have consequences until IFΝγ and/or IL6 cytokine pathway alterations occur and IL6 and IFNγ pathway suffer adverse changes with ageing [43,61];

3. the cytokine network undergoes age-related remodelling [41], variations in pro- or anti-inflammatory cytokines might influence successful ageing and longevity and the multiplex analysis of cytokine levels are useful in defining a successful ageing profile [41,42];

4. the early evolution of immune response is influenced by the positive inter-regulation between production of IFNγ-IL10 and IL6-IL4 cytokines in men, and the negative inter-regulation of IL6-IL10 cytokines in women [43]. Similarly, the late evolution of immune response seems to be influenced by the positive inter-regulation between the production of IFNγ-IL4 in men and by IL6-IFNγ in women. These cytokine relationships are “dual gender specific biomarkers” that could well be used to develop more specific approaches.

These independent findings support a new perspective of research: the comprehension of how the different gender pathways interfere with ageing and lead to diseases, and how they interfere with the success of current therapies, is of utmost importance in translational medicine physiological treatment. The above mentioned gender-specific Th cytokine pathways and the “dual gender specific cytokines” are peripheral blood ageing gender-specific targets and biomarkers and would open up an interesting field for research in human health and disease.

Indeed, important points support the validity of our hypothesis and feasibility of this new research perspective, looking for ageing gender specific biomarkers that can be identified from the general ageing population.

One of the biggest problems in finding good biomarkers of aging is that so many measures are indicators of disease rather than measures of "normal" function [62]. Some gerontologists believe that ageing is not a process or set of processes, but rather is the cumulative effect of damage that makes the body unable to response to external stimuli, others believe that ageing is itself a disease. Searching for biomarkers of ageing only makes sense if there really are relevant biological processes to be measured [62].

The process of ageing is a complex phenomenon, it is the consequence of the deterioration of more than one system [62,63]. Ageing, in the biological sense, is the loss of the ability to maintain homeostasis, that means the loss of the ability to respond to environmental challenges, such as heat or cold or infection, by a) overcoming the challenge and b) restoring normal function. Loss of homeostatic ability can occur at the level of the whole organism or in one or more of its parts.

This assumption leads to the conviction that a “panel” of biomarkers that reflects the condition of an array of critical systems is needed in order to assess the biological age of any organism. Therefore, a specific “panel” could be useful if the biomarkers are predictive of the deterioration in multiple systems. A “panel” of such biomarkers would allow predictions of health span that might be quite different from lifespan.

A low-grade systemic inflammation characterizes ageing and this pro-inflammatory status underlies biological mechanisms responsible for age-related inflammatory diseases [64]. On the other hand, clinical and epidemiological studies show a strong association between chronic infection, inflammation and age-related disease. A wide range of factors, including smoking, infections, obesity, genetics and declining of sex hormone levels may contribute to systemic low-grade inflammatory activity in older individuals [65]. Progressive increase of mean age and life expectancy parallels, in fact, an increment of chronic inflammation which are an index of risk for chronic degenerative diseases such as cancer, cardiovascular, autoimmune or neurodegenerative diseases among the elderly. The goal must be to identify non-symptomatic individuals with high susceptibility to the disease, which can benefit from protocols for the prevention or early intervention.

Therefore, predictive medicine must 1) as a first step anticipate the deleterious effect of chronic degenerative diseases using markers to identify people with pro-inflammatory status and 2) then use high risk markers to predict the developing of a specific chronic degenerative disease before the clinical manifestation. This innovative approach may offer substantial advantages, since the promise of personalized medicine is to preserve individual health in people with high risk by starting early treatment or prevention protocols [1].

On the basis of the above considerations a feasible plan, to identify predictive and diagnostic biomarkers for physiological ageing and age linked diseases, can be identified by using as first step a) the immunological “panel” of “dual gender biomarkers” and then b) the high risk markers for developing a specific chronic degenerative disease, generated by molecular, genetic and neuroimaging techniques.

The use of the immunological “panel” of “dual gender biomarkers” as first approach to identify people with pro-inflammatory status is supported by the following reasons:

1. The levels of “dual gender-specific cytokines” are dual-biomarkers of gender-specific Th cytokine pathways. They are measurable “non-disease related aging biomarkers”, useful to indicate if the subject conditions comply with an healthy state or with a transition to an inflammatory state. These biomarkers select the population according to gender that, as we reported, is the main biomarker for biological and functional differences in the immune response, susceptibility to disease and therapeutic response. “Dual gender biomarkers” represent gender specific biomarkers for homeostasis in the immune response and the human longevity is directly correlated with the optimal functioning of the immune system.

2. The gender-specific Th cytokine pathways are gender-specific target underlying healthy biological processes, that can be related to relevant functions and to be measured in healthy people.

3. Cytokines are easily measurable biomarkers, as they reside in peripheral blood .

4. These immunological biomarkers are predictive of the deterioration in multiple systems that would allow predictions of health span that might be quite different from lifespan.

**Figure 6:**
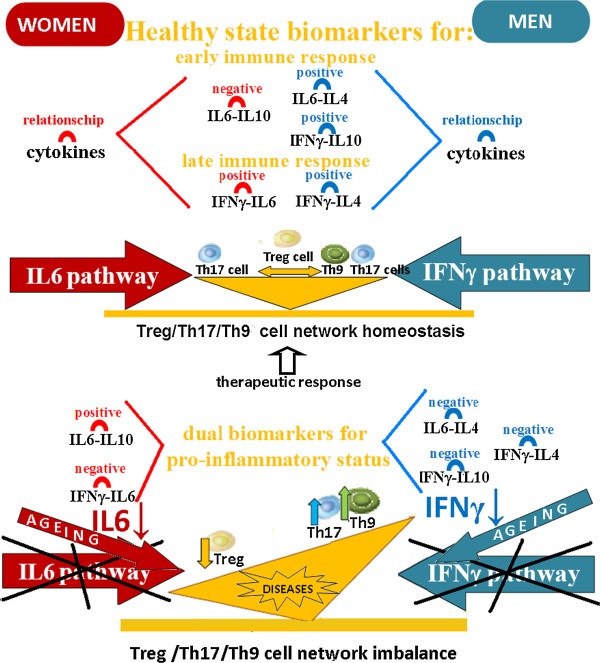
**The normal level of "dual gender specific cytokines" in all “dual gender biomarkers”, is an index for an healthy state because it is an indicator of the ability to maintain physiological homeostasis.** Level variation of "dual gender specific cytokines" (for example IFNγ-IL10, IL6-IL4 and IFNγ-IL4 in men) in a direction that agrees with the positive or negative sign of their healthy relationship (in this case positive), it is an index for a transient inflammation, because it is still an indicator of immune system homeostasis. Level variation of "dual gender specific cytokines" in a direction that does not agree (in this case negative) with the positive or negative sign of their healthy relationship, it is index for a chronic inflammation, because it is an indicator of loss of homeostatic ability.

With this evaluation system, the normal level of "dual gender specific cytokines" in all “dual gender biomarkers”, is an index for an healthy state because it is an indicator of the ability to maintain physiological homeostasis (Figure 5). Level variation of "dual gender specific cytokines" (for example IFNγ-IL10, IL6-IL4 and IFNγ-IL4 in men) in a direction that agrees with the positive or negative sign of their healthy relationship (in this case positive), it is an index for a transient inflammation, because it is still an indicator of immune system homeostasis; the level variation of "dual gender specific cytokines" in a direction that does not agree (in this case negative) with the positive or negative sign of their healthy relationship, it is index for a chronic inflammation, because it is an indicator of loss of homeostatic ability (Figure 6).

These new “panel” of biomarkers, predictive for a) the inflammation state and b) the specific type of disease involved, may lead to reduces diseases incidence rate and to distinguish clinical subtypes of a single disease to better tailor both, potential prevention strategies and/or early intervention protocols for chronic degenerative diseases, maintaining an acceptable quality of life.

Further study will validate or refute this new hypothesis. Operative proposals for the heath care systems are now needed to verify potential benefits in predictive medicine.

## Competing interests

The authors declare that they have no competing interests.

## Authors’ contributions

AMB designed the research, analyzed and interpreted data, wrote the manuscript. IC and PP contributed to design the research and analyze data; TDB contributed to analyze data. All authors read and approved the final manuscript.
